# Efficacy of adjuvant chemotherapy with carboplatin for early triple negative breast cancer: a single center experience

**DOI:** 10.18632/oncotarget.18118

**Published:** 2017-05-23

**Authors:** Marcus Vetter, Spyridon Fokas, Ewelina Biskup, Thomas Schmid, Fabienne Schwab, Andreas Schoetzau, Uwe Güth, Christoph Rochlitz, Rosanna Zanetti-Dällenbach

**Affiliations:** ^1^ Department of Medical Oncology, University Hospital Basel, Basel, Switzerland; ^2^ Women’s Hospital,University Hospital Basel, Basel, Switzerland; ^3^ Department of Internal Medicine, University Hospital Basel, Basel, Switzerland; ^4^ The Royal Marsden Hospital, London, United Kingdom; ^5^ Brust-Zentrum Zurich, Zurich, Switzerland; ^6^ Sankt Claraspital, Basel, Switzerland; ^7^ Shanghai University of Medicine and Health Sciences, Department of Basic Medical College, Shanghai, PR China

**Keywords:** early breast cancer, triple negative breast cancer, chemotherapy, carboplatin, platinum-based chemotherapy

## Abstract

**Background:**

Anthracycline- and taxane-based adjuvant chemotherapies are the most frequently used systemic treatments for women with triple negative breast cancer (TNBC). Adding platinum derivatives in the neo-adjuvant setting has been shown to not only improve the pCR rates, but also the 3 year DFS for TNBC patients; however, data on platinum derivatives in the adjuvant setting are limited.

**Methods:**

We conducted a retrospective, single-center study in a Swiss breast cancer cohort to evaluate the role of carboplatin in addition to standard adjuvant therapy (anthracyclines and/ or taxanes) in early TNBC patients. All patients with stage I-III TNBC who underwent primary breast surgery between 2004 and 2014 were included.

**Results:**

Eighty-three patients were included in the analysis. Stage and grade were well balanced between patients treated with standard chemotherapy (*N*=54; cohort A) or standard chemotherapy plus carboplatin (*N*=29; cohort B). The median time to local relapse (LRFS) was 15.0 months in cohort A *versus* 16.0 months in cohort B (*p*=0.655). The median time to distant relapse (DRFS) was 29.5 months in cohort A *versus* 25.0 months in cohort B (*p*=0.606) There was also no difference in overall survival between the two cohorts (mean overall survival 98 and 91 months, respectively; *p*=0.208).

**Discussion:**

Our data suggest that in an unselected cohort of early TNBC patients, the addition of carboplatin in the adjuvant setting may not be beneficial with respect to relapse-free and overall survival. Further prospective trials to evaluate the addition of platinum in the adjuvant setting are warranted, especially to define subgroups of TNBC patients, which might benefit from carboplatin therapy.

## INTRODUCTION

Approximately 10-20% of women with invasive breast cancer have tumors that are histopathologically negative for the estrogen receptor (ER), progesterone receptor (PR), and the human epidermal growth factor receptor 2 (HER-2) [1-3]. These tumors, termed ‘triple-negative breast cancer’ (TNBC), represent a particularly aggressive form of the disease. Women with TNBC tend to be younger with more advanced disease at diagnosis than women with other types of breast cancer and have a shorter median time to relapse, a higher rate of relapse in the first two years (15%), and a worse prognosis [4, 5].Prognosis is particularly poor once metastases have developed and median overall survival (OS) for women with metastatic TNBC is less than 12 months [6] [7].

Treatment of TNBC is challenging as, by definition, patients with TNBC do not respond to endocrine therapy or agents that target the HER-2 pathway [8] [9].Targeted therapies against the epidermal growth factor receptor and the vascular endothelial growth factor receptor also appear to be ineffective in unselected patients with TNBC [10] [8]. An effective targeted therapy for TNBC remains a major unmet clinical need. Several novel therapies are being investigated in phase II and phase III trials, such as poly(ADP-ribose) polymerase inhibitors (PARP-Inhibitors) for BRCA-mutated TNBC, antiandrogens for androgen receptor (AR)-positive TNBC, and gamma-secretase inhibitors for TNBC with mutations in the PEST domain of NOTCH proteins [8].

Triple-negative breast cancer comprises a heterogeneous breast cancer subtype and this heterogeneity may explain the lack of success of targeted therapies in unselected patients [8, 11]. Up until 2011, there were several subtypes of TNBC, including basal-like (BL1 and BL2), immunomodulatory (IM), mesenchymal, mesenchymal stem-like (MSL), and luminal androgen receptor subtypes [11, 12]. In 2016, the same group refined the initial “Vanderbilt” classification: the subtypes MSL and IM seemed to be contributed to tumor infiltrating lymphocytes and tumor stromal cells, thus resulting in overall 4 subtypes. [13] Up until recently, the subtype of TNBC has no impact on clinical treatment decisions [14] [15] [16]. The breakthrough have been achieved by Gepar Sixto Trial, which reported a significant improvement of the 3 years DFS in TNBC patients. [17]

The standard treatment for TNBC includes surgery, cytotoxic chemotherapy, and radiotherapy. Anthracyclines and taxanes are the most active cytotoxic agents for TNBC [18-20]. In the neoadjuvant setting, anthracyclines achieve relatively high pathological complete response (pCR) rates in women with TNBC (27% in one study [7]). More recently, several studies have reported promising data with neoadjuvant platinum-based regimens in TNBC [20]. Neoadjuvant paclitaxel plus carboplatin and liposomal doxorubicin achieved a very high pCR rate (60%) in women with TNBC in the CALGB 40603 trial [21]. Similarly, in the GeparSixto trial the addition of carboplatin to neoadjuvant doxorubicin plus bevacizumab plus paclitaxel therapy resulted in a total (ypT0 and ypN0) pCR rate of 53.2% in TNBC [22]. The pCR rate was higher (61.5%) in women with TNBC who carried the germline mutation in BRCA [23].Women with TNBC appear to be particularly sensitive to platinum-based chemotherapy, thus it is suggestive that particularly the BRCA mutated subgroup of TNBC should receive platinum derivatives as part of their neoadjuvant (and/or adjuvant) treatment. [17] [24] A retrospective analysis of women with breast cancer (both local and advanced) treated with platinum-based chemotherapy showed that pCR rates were significantly higher in women with TNBC compared with other types of breast cancer (88% *versus* 51%, respectively; *p* = 0.005) [25]. However, this did not translate into improved OS which was shorter for women with TNBC compared with other types of breast cancer.

Evidence supporting the use of adjuvant chemotherapy in TNBC is scarce and based mostly on post-hoc subgroup analyses of trials [26]. Addition of taxanes improves disease-free survival and OS in TNBC compared with non-taxane based regimens [27] [28]. In the GEICAM 9805 study and the Breast Cancer International Research Group (BCIRG) 001 study, adjuvant docetaxel, doxorubicin and cyclophosphamide was superior to fluorouracil, doxorubicin and cyclophosphamide [29, 30]. There is a consensus that patients with TNBC should undergo adjuvant chemotherapy to reduce relapse rate and to improve OS [14] [15]; however, the optimal adjuvant regimen has not been determined. Data on adjuvant and postoperative use of carboplatin in TNBC are elusive.

To date, administration of adjuvant carboplatin for TNBC is not common in Switzerland, mainly because most of the TNBC patients receive a neoadjuvant induction therapy with carboplatin. However, since the first data showing the value of neoadjuvant carboplatin in TNBC were published, adjuvant carboplatin has been routinely used in the treatment of women with TNBC at our center. In this study, we retrospectively investigated whether the addition of adjuvant carboplatin to standard therapy was beneficial in an unselected TNBC population.

## PATIENTS AND METHODS

This was a retrospective single-centre database study. Data were extracted from the Basel Breast Cancer Database, which includes all newly diagnosed primary invasive breast cancer cases treated at the University Hospital Basel, Switzerland since 1990. Additional data were obtained from chart review from the department of Medical Oncology, University Hospital Basel. The local ethics committee of the University Hospital Basel approved this research project.

Data from all patients diagnosed with early TNBC (Stage I-III) from 2004 until 2014 were collected. The main inclusion criteria for our analysis were: confirmed histopathological diagnosis of TNBC, defined by negative immunohistochemistry for ER, PR, and HER2 in accordance to American Society of Clinical Oncology/College of American Pathologists; Stage I-III disease; age between 18-99 years; surgery and radiotherapy according to local tumor board/guidelines; and at least one cycle of cytotoxic chemotherapy for localized TNBC. Two independent breast pathologists determined all histopathologic results for this study. Patients were followed until December 2014 or until relapse and/or death.

### Clinicopathological data

The following data were obtained for all patients: age at initial diagnosis, histological subtype, grading, hormonal receptor and HER2 status, tumor stage according to Union for International Cancer Control (UICC), and tumor-node-metastases (TNM) classification [31] [32]. Because HER2 status has been routinely assessed for all patients since 2004, we included data from 2004 to 2014 in the analysis. All data for the prescribed chemotherapies were available, including type of drugs and number of cycles. Toxicity and quality of life data were not collected and analyzed in this study because data obtained from the retrospective chart review were not considered to be comprehensive enough to analyze.

### Chemotherapy regimes

All included patients received either standard of care adjuvant anthracycline / taxane-based chemotherapy (cohort A) or standard chemotherapy with additional adjuvant carboplatin-based chemotherapy (cohort B). All treatment decisions were based on an interdisciplinary weekly breast cancer tumor board. Chemotherapy regimens were selected by the breast oncologist in charge and in accordance with international guidelines from the European Society for Medical Oncology and the National Comprehensive Cancer Network [15] [14]. Patients who had neoadjuvant or palliative therapy approaches were excluded from this study.

Patients in cohort A received one of the following established anthracycline and /or taxane-based regimes: four cycles of cyclophosphamide plus either epirubicin or adriblastine followed by docetaxel, or paclitaxel; or four cycles of cyclophosphamide plus either epirubicin or adriblastine; or four cycles of cyclophosphamide plus docetaxel. Patients in cohort B received four cycles of cyclophosphamide plus either epirubicin or adriblastine followed by four cycles of carboplatin plus docetaxel.

### Demographic data

Overall, 83 patients were included, with a median age of primary diagnosis at 52 years, with a median follow up time of 59 months. 54 were included in cohort A, with an age range from 26-82 years of age (median age at diagnosis 52 years), with a median follow up time of 84 months. Cohort B included 29 patients; with a median age of diagnosis 53 years (38-78 years of age) and a median follow up time of 36months. Further details are listed in Table 1. Both white Caucasian (predominantly) and other races were included.

**Table 1:** Baseline characteristics for patients with triple negative breast cancer treated with standard chemotherapy or standard chemotherapy plus carboplatin

	All (*N* = 83)	Cohort A (standard chemotherapy) *N* = 54	Cohort B (carboplatin-based chemotherapy) *N* = 29	*p* value
Median age (range), years	52 (26–82)	51 (26.0–82.0)	53 (38.0–71.0)	0.782
Median follow up (range), months	59 (1–146)	84 (1.0–146.0)	36 (12.0–72.0)	<0.001
Histopathologic subtype, *n* (%)				0.979
Invasive ductal	68 (81.9)	42 (77. 8)	26 (89.7)	
Lobular	4 (4.8)	3 (5.6)	1 (3.5)	
Ductul lobular	1 (1.2)	1 (1.9)	0 (0.0)	
Medullar	6 (7.2)	4 (7.4)	2 (6.9)	
Papillary	1 (1.2)	1 (1.9)	0 (0.0)	
M.Paget	2 (2.4)	2 (3.7)	0 (0.0)	
Others	1 (1.2)	1 (1.85)	0 (0.0)	
Grade				0.565
1	4 (4.8)	2 (3.7)	2 (6.9)	
2	16 (19.3)	12 (22.2)	4 (13.8)	
3	62 (74.7)	39 (72.2)	23 (79.3)	
Missing	1 (1.2)	1 (1.9)	0 (0.0)	
Stage AJCC				0.459
I	34 (41.0)	22 (40.7)	12 (41.4)	
II	44 (53.0)	30 (55.6)	14 (48.3)	
III	5 (6.0)	2 (3.7)	3 (10.3)	
Chemotherapy cycles, *n* (%)				<0.001
<4	6 (7.2)	3 (5.6)	3 (10.3)	
4–5	27 (32.5)	27 (50.0)	0 (0.0)	
6–7	27 (32.5)	21 (38.9)	6 (20.7)	
≥8	22 (26.5)	2 (3.7)	20 (69.0)	
Unknown	1 (1.2)	1 (1.9)	0 (0.0)	

AJCC, American Joint Committee on Cancer

**Table 2:** Local and distant relapse-free survival and overall survival for patients with triple negative breast cancer treated with standard chemotherapy or standard chemotherapy plus carboplatin

	All (*N* = 83)	Cohort A (standard chemotherapy) *N* = 54	Cohort B (carboplatin-based chemotherapy) *N* = 29	*p* value
Relapse, *n* (%)				
No relapse	74 (89.2)	48 (88.9)	26 (89.7)	1.000
Relapse	9 (10.8)	6 (11.1)	3 (10.3)	
LRFS, months				
Mean (±SD)	16.5 (±3.87)	16.7 (±4.73)	16.0 (NA?)	NA
Median (range)	15.5 (13.0–22.0)	15 (13.0–22.0)	16 (16.0–16.0)	0.655
DRFS, months				
Mean (±SD)	36.9 (±33.4)	44.2 (±39.8)	22.3 (±5.51)	0.241
Median (range)	25.0 (13.0–119.0)	29.5 (13.0–119.0)	25.0 (16.0–26.0)	0.606
Time to relapse, months				
Mean (±SD)	31.7 (± 33.8)	36.3 (±41.7)	22.3 (±5.5)	0.453
Median (range)	19.0 (13.0–119)	17.0 (13.0–119.0)	25.0 (16.0–26.0)	0.604
Death, *n* (%)				
No	70 (84.3)	46 (85.2)	24 (82.8)	1.000
Yes	13 (15.7)	8 (14.8)	5 (17.2)	
Time to death, months				
Mean (±SD)	37.8 (±29.2)	44.6 (±35.5)	26.8 (± 10.2)	0.216
Median (range)	35.0 (4.0–120.0)	40.5 (4.0–120)	30.0 (12.0–36.0)	0.272

DRFS, distant relapse-free survival; LRFS, local relapse-free survival

### Statistical analysis

The Kaplan-Meier method was used to compare time to relapse (local or metastatic) and overall survival. Differences between cohorts were analyzed using the log rank test. The time to relapse was calculated from the date of diagnosis until first localized or metastatic relapse or to date of last follow-up (for patients without a relapse). Overall survival was calculated from the date of diagnosis until death, or to date of last follow-up, respectively.

The nonparametric Wilcoxon-Test was used to compare ordinary variables between the two groups. Comparisons between nominal parameters were made with Fisher’s exact test. Because the number of events was small, multivariable cox regression including variables such as age, stage or subtype was not performed. In all statistical tests, the level of significance was *p* < 0.05. All evaluations were performed with R Development Core Team software, version 13.1.

## RESULTS

Between January 2004 and December 2014, 134 patients with early TNBC were identified. Fifty-one patients were excluded because patients did not receive any chemotherapy (*N* = 28), received preoperative (neoadjuvant) chemotherapy (*N* = 10), or received their chemotherapy at different hospitals with no available follow-up (*N* = 13). The final analysis population comprised 83 patients with median follow-up of 59 months.

Fifty-four (65.1%) patients were treated with anthracyclines and /or taxanes (cohort A) and 29 (34.9%) patients were treated with anthracyclines and taxanes plus carboplatin-based chemotherapy (cohort B).

The median follow-up was 59 months (range 1-146 months) overall, 84 months in cohort A (range 1-146 months), and 36 months in cohort B (range 12-72 months).

Baseline patient and tumor characteristics (subtype, grading, and stage) did not differ statistically between the two cohorts (Table 1). The median age of the complete cohort was 52 years; overall 81.9% of tumors were invasive ductal carcinoma; 41%were stage I, 53% were stage II, and 6% were stage III; and 74.7% of the tumors were poorly differentiated (Grade 3).

### Treatment

The majority of patients in cohort A (94.44%) received at least four cycles of docetaxel or epirubicin-based chemotherapy (epirubicin/cyclophosphamide *N* = 24; adriblastine/cyclophosphamide *N* = 1; cyclophosphamide/methotrexate/5FU *N* = 1; 5FU/epirubicin/cyclophosphamide *N* = 16; docetaxel/adriblastine/cyclophosphamide *N* = 7; *N* = 1, docetaxel/cyclophosphamide *N* = 3; epirubicin/cyclophosphamide followed by docetaxel *N* = 1). The majority (69%) of patients in cohort B received four cycles of epirubicin/cyclophosphamide followed by four cycles of carboplatin/docetaxel.

The mean number of chemotherapy cycles per patient was 4.7 for patients treated without carboplatin-based chemotherapy (cohort A; 252 cycles in total) and 7.0 for patients treated with carboplatin-based chemotherapy (cohort B; 456 cycles in total). This was due to the additional adjuvant carboplatin-based treatment for patients in cohort B, which resulted in these patients receiving a significantly higher number of adjuvant chemotherapy cycles: eight cycles of chemotherapy were given in 69.0% of patients in cohort B compared with only 3.7% of patients in cohort A (*p* < 0.001).

### Efficacy analysis

**Figure 1:**
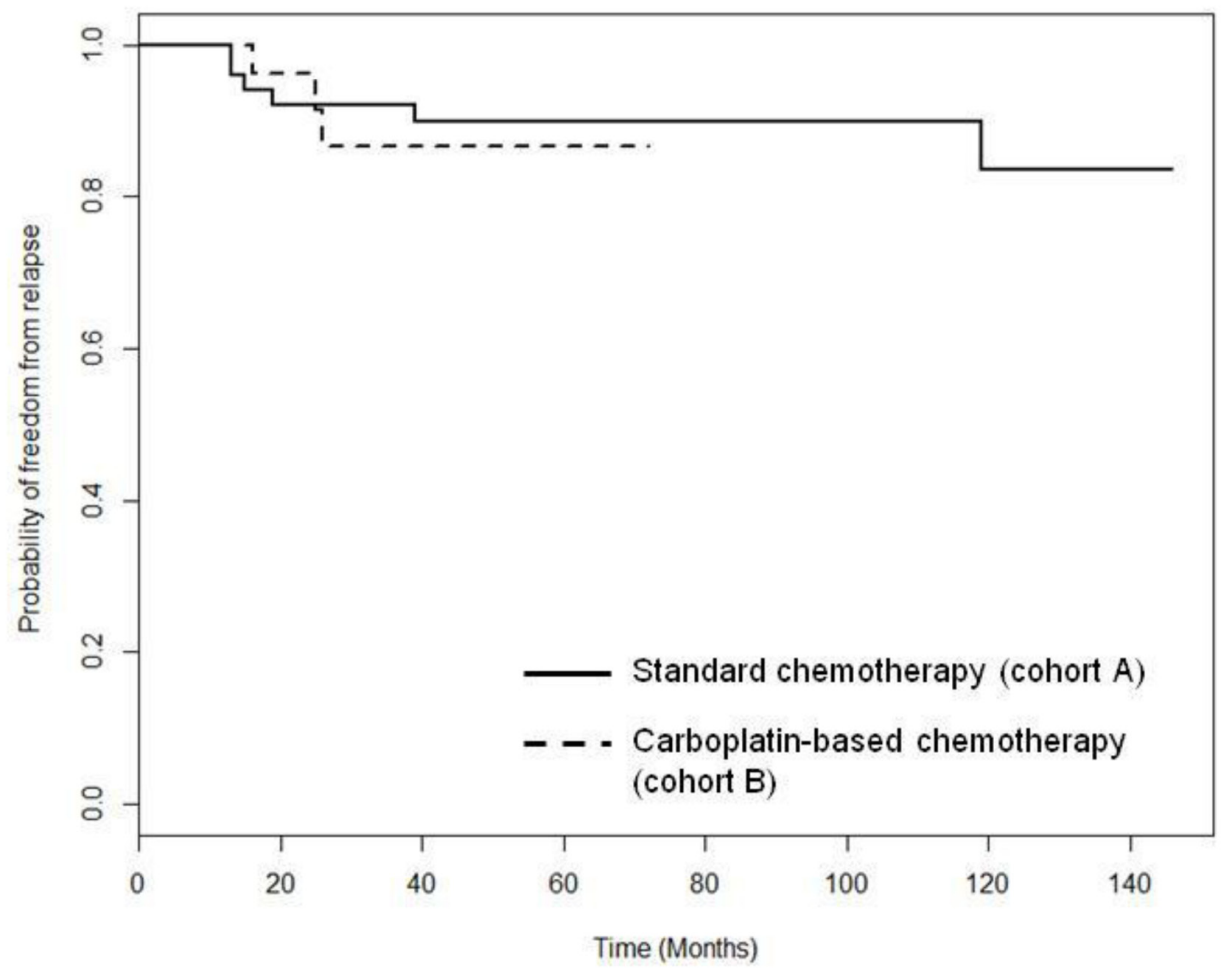
Relapse-free survival for patients with TNBC receiving standard chemotherapy (solid line) or standard chemotherapy plus carboplatin (dashed line) Chisq = 0.1 on 1 degrees of freedom, *p* = 0.755.

Relapses were recorded in nine (10.8 %) patients during follow-up: six (11.1%) patients in cohort A and three (10.3 %) patients in cohort B. The median time to local relapse (LRFS) was 15.0 months in cohort A *versus* 16.0 months in cohort B (*p* = 0.655). The median time to distant relapse (DRFS) was 29.5 months in cohort A *versus* 25.0 months in cohort B (*p* = 0.606The median overall relapse-free survival was not reached in both cohorts. At five years, the overall relapse-free survival rate was 85% in cohort B and 90% in cohort A. (Figure 1).

**Figure 2:**
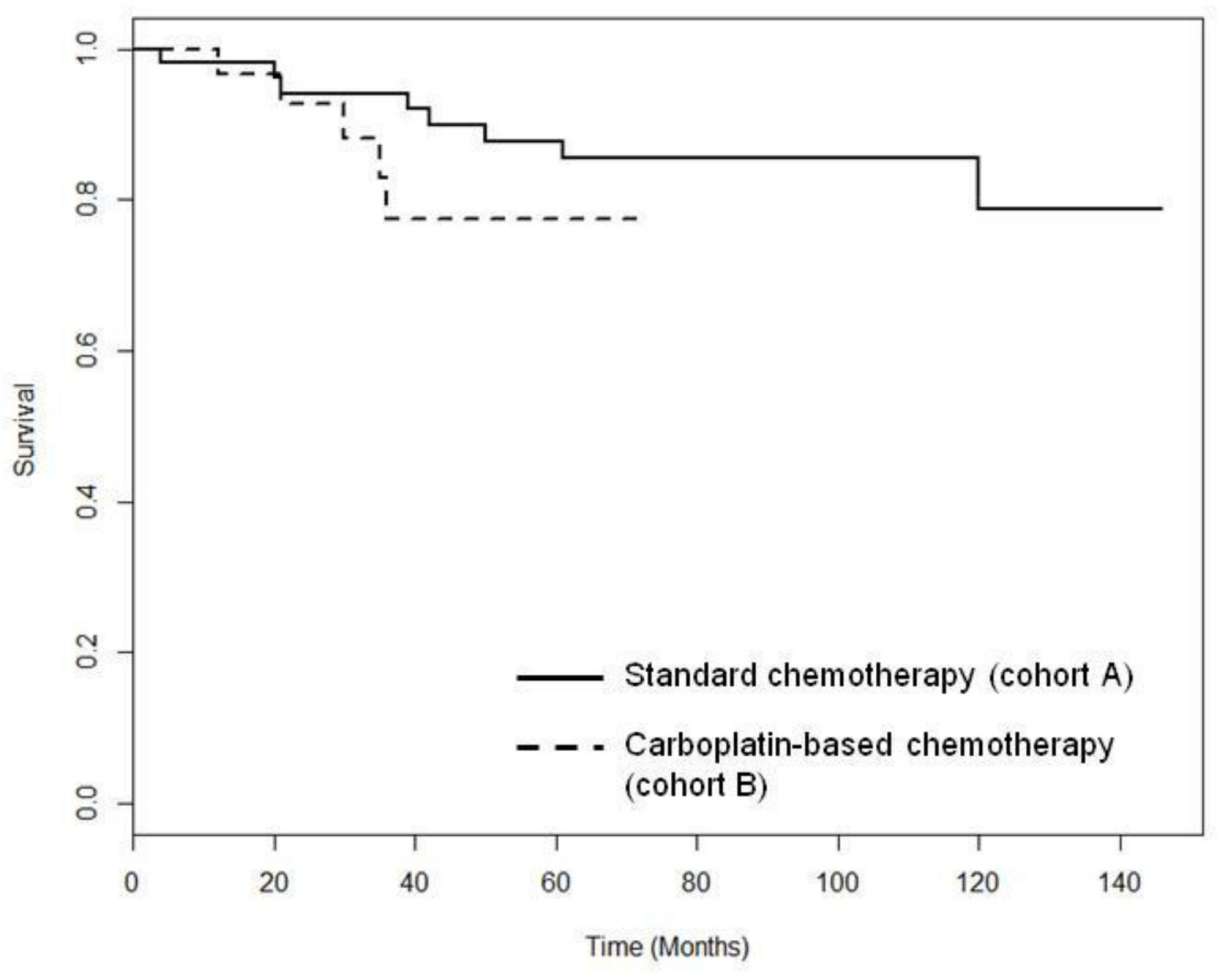
Overallsurvival for patients with TNBC receiving standard chemotherapy (solid line) or standard chemotherapy plus carboplatin (dashed line) Chisq = 1.6 on 1 degrees of freedom, *p* = 0.208.

Thirteen (15.7%) patients died during follow up, eight (14.8%) in cohort A, and five (17.2%)in cohort B. The median time to death overall was 35 months (range 4.0-120.0 months) and was not significantly different for patients treated with anthracycline/taxane-based chemotherapy alone (cohort A: median 40.5 months; range 4.0-120.0 months) or with carboplatin-based chemotherapy (cohort B: median 30.0 months; range 12.0-36.0 months; *p* = 0.272 At five years, the OS rate was 78% in cohort B and 86% in cohort A. (Figure 2). No treatment related deaths were observed.

## DISCUSSION

We retrospectively analyzed efficacy outcomes in women with TNBC treated at Basel University Hospital over a 10-year period who received standard anthracycline- and/or taxane-based chemotherapy with or without carboplatin-based chemotherapy. In our cohort, the addition of carboplatin regimens to standard care did not improve relapse-free survival or OS compared with standard chemotherapy alone. This was in spite of the fact that the patients in the carboplatin group received significantly more therapy, with a median of seven cycles compared with median of 4.7 cycles in the standard chemotherapy group.

A strength of our study was the relatively long follow-up period (median 59 months overall; range 1-146 months). Both relapse-free survival and OS for the TNBC patients from our cohort were better than those reported in older studies with larger cohorts [33] [34] [35] [36] [37]. In our study, five-year relapse-free survival was over 80% in both groups treated with (cohort B 90%) and without carboplatin (cohort A 85%) (Figure1). This compares well to historical data that reported five-year and ten-years relapse-free survival rates of 60 to 80% with taxanes-based chemotherapy including dose dense regimes. [38] [39], 65-85% with anthracycline- and taxanes-based therapy including dose dense regimes [40] and 83.7% anthracycline based chemotherapy plus bevacizumab (BEATRICE Study). [10] Similarly, the five-year OS rate in our cohort (78% and 86%) (Figure 2) was higher than the rates previously reported. [33] [34] [35] [36] [37] But comparable to newer 2^nd^ and 3^rd^ generation regimes. [29] [22] One reason for the better outcome in our cohort might be the implementation of more extensive, longer and improved treatment options (surgery, radiotherapy and systemic therapy)in recent decades [41] [42]. More than 58% of patients received six or more cycles of treatment. Data from Celloni et al. demonstrated that patients with ER-negative tumors might benefit from longer chemotherapy with cyclophosphamide, methotrexate and 5FU [43]. But in contrast newer maintenance data are showing some benefit for extended treatment in TNBC, in particular in high risk patients. [44] An extensive use of supportive care might also have contributed to better outcomes. [45]

A clear benefit of adjuvant carboplatin use in TNBC is still controversial. Data from a number of trials support the value of neoadjuvant platinum-based chemotherapy in women with TNBC [21] [23] [46]. One might assume that the efficiency of adjuvant platinum-based regimens would be comparable to that seen in the neoadjuvant setting; however, this has yet to be conclusively demonstrated. Older studies in general breast cancer have shown that preoperative chemotherapy had the same outcome as adjuvant chemotherapy [47] [48]. Further data showing a benefit of adjuvant platinum-based chemotherapy is needed before these regimens can be recommended in women with TNBC to prevent unnecessary toxicities. An ongoing Phase III Trial of carboplatin as adjuvant chemotherapy *vs*. observation (POST-Neo Adjuvant Study) might provide more insights on whether carboplatin is effective in TNBC.

Tumor stage and biology might be crucial factors to determine outcomes and response of the chemotherapy in TNBC. Both stage and histological subtype were equally distributed between cohorts (Table 1). The biologic subtypes, including AR+ and immunophenotypes, were not available as they are not routinely determined in our departments. Treatment of TNBC is challenging as, by definition, patients with TNBC do not respond to endocrine therapy or agents that target the HER-2 pathway [8] [9]. Targeted therapies against the epidermal growth factor receptor and the vascular endothelial growth factor receptor also appear to be ineffective in unselected patients with TNBC [10] [8]. An effective targeted therapy for TNBC remains a major unmet clinical need. Several novel therapies are being investigated in phase II and phase III trials, such as poly(ADP-ribose) polymerase inhibitors (PARP-Inhibitors) for BRCA-mutated TNBC, antiandrogens for androgen receptor (AR)-positive TNBC, and gamma-secretase inhibitors for TNBC with mutations in the PEST domain of NOTCH proteins [8].

The presence of BRCA 1/2 mutations appears to be a positive selection criteria for the use of platinum-based regimens. Expression of BRCA1/2 mutations are thus far the sole biomarkers to identify TNBC patients that would respond to carboplatin therapy. Women with TNBC who have metastatic disease and/or BRCA germline mutations appear to benefit the most from platinum-based regimens [49, 50] [25] [51] [52, 53]. The TNT study of metastatic or recurrent locally advanced TNBC or BRCA 1/2 mutated breast cancer showed that taxanes and carboplatin were comparably well tolerated in both groups, and that carboplatin was beneficial in BRCA1/2 mutated subgroup [51]. Byrski et al. reported that platinum-based chemotherapy was effective in a high proportion of patients with BRCA1-associated early stage invasive breast cancer with pCR rates of 61%. [54] Telli et al. also showed a favorable pCR with carboplatin andiniparib (a PARP-inhibitor) in patients with TNBC or BRCA 1/2 mutated breast cancer [55]. However, this study also demonstrated a benefit for carboplatin and iniparib in TNBC patients who did not have BRCA1/2 mutations but who did have BRCA 1/2 abnormalities detected by the homologous recombination deficiency loss of heterozygosity assay [55]. This suggests that even patients without BRCA mutations detected by standard sequencing methods benefit from carboplatin-based regimens. BRCA 1/2 status was not available for the patients in our study since regular testing of BRCA 1/2 did not begin in our unit until 2014. After consideration, we decided not to retest stored histological samples for BRCA 1/2 mutation status because the number of events was too low to draw representative statistical conclusions. Nevertheless, further investigations are warranted since BRCA1-mutation carriers account for 10-20% of TNBC [56] and sporadic TNBC shares many pathologic and clinical features with BRCA 1-associated breast cancer [57].

There are several limitations to our study. First, this was a retrospective analysis. The study was unicentric and focused on Swiss patients only. The population of TNBC patients included was relatively small due to our selection criteria. Furthermore, the number of events (relapses and deaths) in both cohorts was low which limited the statistical power of our analyses.

In conclusion, our retrospective analysis did not show a benefit of adding adjuvant carboplatin-based chemotherapy to standard of care adjuvant anthracycline / taxane-based chemotherapy in women with early TNBC. However, this retrospective analysis is hypothesis-generating at best. Based on our findings, we suggest that in an unselected group of patients with early TNBC, carboplatin should be used with caution and always with an individual approach according to patient’s general status, age at diagnosis, comorbidities, BRCA 1/2 mutation status, and precedent therapies.

## Abbreviations

AR: androgen receptor
BL: basal-like
ER: estrogen receptor
HER2: human epidermal growth factor receptor 2
OS: overall survival
PARP: poly(ADP-ribose) polymerase
pCR: pathological complete response
PR: progesterone receptor
TNBC: triple-negative breast cancer
